# A signature based on glycosyltransferase genes provides a promising tool for the prediction of prognosis and immunotherapy responsiveness in ovarian cancer

**DOI:** 10.1186/s13048-022-01088-9

**Published:** 2023-01-07

**Authors:** Xuyao Xu, Yue Wu, Genmei Jia, Qiaoying Zhu, Dake Li, Kaipeng Xie

**Affiliations:** 1 Department of Gynecology, Nanjing Maternity and Child Health Care Hospital, Women’s Hospital of Nanjing Medical University, Nanjing, 210004 China; 2 Department of Women Health Care, Nanjing Maternity and Child Health Care Hospital, Women’s Hospital of Nanjing Medical University, Nanjing, 210004 China; 3 Department of Public Health, Nanjing Maternity and Child Health Care Hospital, Women’s Hospital of Nanjing Medical University, Nanjing, 210004 China

**Keywords:** Ovarian cancer, Glycosyltransferase, Prognosis, Immunotherapy

## Abstract

**Background:**

Ovarian cancer (OC) is the most fatal gynaecological malignancy and has a poor prognosis. Glycosylation, the biosynthetic process that depends on specific glycosyltransferases (GTs), has recently attracted increasing importance due to the vital role it plays in cancer. In this study, we aimed to determine whether OC patients could be stratified by glycosyltransferase gene profiles to better predict the prognosis and efficiency of immune checkpoint blockade therapies (ICBs).

**Methods:**

We retrieved transcriptome data across 420 OC and 88 normal tissue samples using The Cancer Genome Atlas (TCGA) and Genotype-Tissue Expression (GTEx) databases, respectively. An external validation dataset containing 185 OC samples was downloaded from the Gene Expression Omnibus (GEO) database. Knockdown and pathway prediction of B4GALT5 were conducted to investigate the function and mechanism of B4GALT5 in OC proliferation, migration and invasion.

**Results:**

A total of 50 differentially expressed GT genes were identified between OC and normal ovarian tissues. Two clusters were stratified by operating consensus clustering, but no significant prognostic value was observed. By applying the least absolute shrinkage and selection operator (LASSO) Cox regression method, a 6-gene signature was built that classified OC patients in the TCGA cohort into a low- or high-risk group. Patients with high scores had a worse prognosis than those with low scores. This risk signature was further validated in an external GEO dataset. Furthermore, the risk score was an independent risk predictor, and a nomogram was created to improve the accuracy of prognostic classification. Notably, the low-risk OC patients exhibited a higher degree of antitumor immune cell infiltration and a superior response to ICBs. B4GALT5, one of six hub genes, was identified as a regulator of proliferation, migration and invasion in OC.

**Conclusion:**

Taken together, we established a reliable GT-gene-based signature to predict prognosis, immune status and identify OC patients who would benefit from ICBs. GT genes might be a promising biomarker for OC progression and a potential therapeutic target for OC.

**Supplementary Information:**

The online version contains supplementary material available at 10.1186/s13048-022-01088-9.

## Introduction

Ovarian cancer (OC) is the most lethal malignancy of the female reproductive system, the incidence of which is only secondary to cervical cancer and uterine corpus cancer. According to the latest global cancer statistics of 2020, ovarian cancer accounts for approximately 3.4% of 9.2 million new cancer cases in females and 4.7% of mortality [1]. Due to the insidious onset of ovarian cancer and lack of effective screening tools, nearly 70% of patients are not diagnosed until the advanced stage (stage III or IV) [2, 3]. The 5-year survival rate of advanced patients is lower than that of patients who are diagnosed at an early stage [4]. Although much progress has been achieved in non-traditional treatment, such as immunotherapy and targeted therapy, the reduction in mortality and recurrence still remains limited [5, 6]. Similar to other malignant tumours, heterogeneity exists widely across subtypes or even within a single tumour, which may result in no response to corresponding treatments [7–9]. Therefore, there is an urgent need to identify novel molecular signatures to predict prognosis and evaluate the sensitive subpopulations of immunotherapy, which contribute to the improvement of treatment success and the development of precision medicine.

Posttranslational modifications (PTMs) of proteins are often dysregulated in cancer. The specific function of a protein is dynamically achieved by the catalytic action of many enzymes involved in PTMs, suggesting that these enzymes may provide clues for cancer research [10, 11]. Glycosylation is one of the most common PTMs of proteins and plays a vital role in many critical biological processes, including cell adhesion, growth, signal transduction and immune response, by affecting the function of modified proteins [12, 13]. The biosynthesis of glycosylation is a complex process orchestrated by several glycosyltransferases and glycosidases. It has been widely recognized that abnormal glycan changes in proteins are involved in many pathological states, such as viral infection, cancer progression and the inflammatory process [14]. Aberrant glycosylation is considered a marker of cancer, the main factor of which is the abnormal expression of glycosyltransferases (GTs) translated from corresponding GT genes [15, 16].

Numerous studies have proven that altered expression levels of GTs can directly affect the malignant phenotypes of cancer, such as proliferation [17], metastasis [18] and drug resistance [16], indicating that targeting GTs may help us understand the role of aberrant glycosylation in cancer pathogenesis [19]. In ovarian cancer, mounting evidence has suggested that GTs played a critical role in its malignant progression. For example, overexpression of sialyltransferase ST3GAL1 was proven to promote progression and paclitaxel resistance in OC [20]. Elevated α1,3-mannosyltransferase 3 (ALG3) was reported to promote peritoneal metastasis of OC through increasing interaction of α1,3-mannosylated uPAR and ADAM8 [21]. Huang et al. demonstrated that glycosyltransferase 8 domain containing 2 confers CDDP (cis-dichlorodiammine-platinum) resistance through the FGFR/PI3K signalling axis [22]. What’s more, a recent study has proved that GALNT14 was significantly upregulated in OC and regulated ferroptosis through the EGFR/mTOR pathway [23]. However, to our knowledge, a systematic analysis of GT genes in OC is still blank. Since GTs could affect the prognosis of OC through induction of malignancy phenotypes, discovering the GT-gene signature for risk stratification is in demand. In addition, it is worth mentioning that glycosylation is not only under the control of epigenetic regulation (DNA methylation, histone acetylation and noncoding RNAs) but also itself is an epigenetic modifier of  histones that participates in cancer progression [24, 25]. Given that cancer cells can hijack various existing epigenetic modifications, including glycosylation, to modulate antitumour immunity and lead to tumour escape, epigenetic signatures appear to be promising candidates for predicting the outcomes of immunotherapy [26]. For example, sialic acid sugars on the surface of cancer cells are recognized as potent immune modulators that contribute to the immunosuppressive microenvironment and tumour immune evasion [27]. Therefore, these studies provide a rationale for the potential ability of GT-signature to predict immunotherapy success, contributing to personalized medicine.

**Fig. 1:**
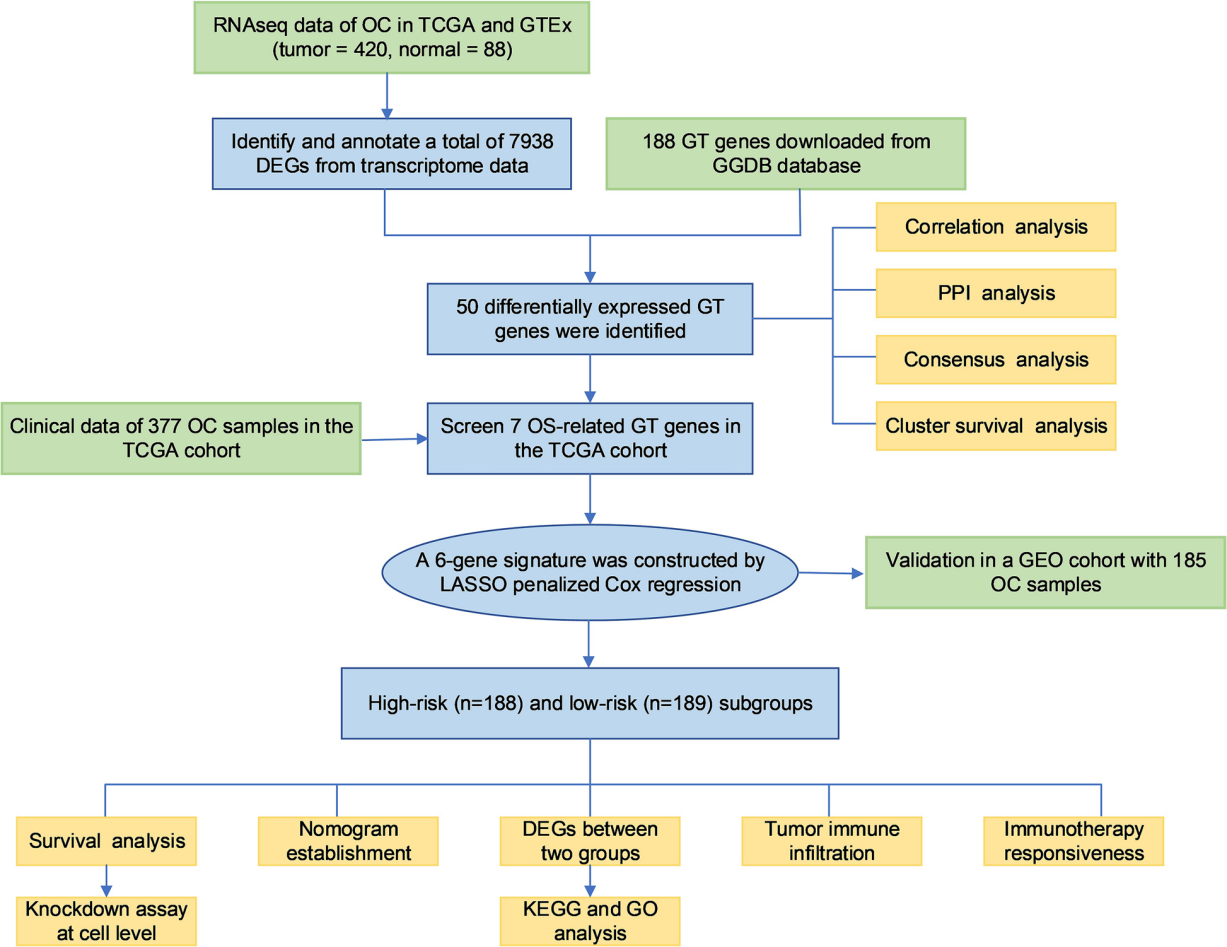
Workflow diagram. The specific workflow graph of our study

In this study, we performed a systematic analysis to determine the expression pattern of glycosyltransferase genes between normal ovarian and OC tissues and determine whether OC could be stratified by these GT gene expression profiles. The flow chart of this study is shown in Fig. 1. By using bioinformatic analysis of the samples from The Cancer Genome Atlas (TCGA) and Genotype-Tissue Expression (GTEx) projects, we established a risk signature of six GT genes that was able to predict the prognosis of OC and was further validated in the GEO database. We then studied the correlation between this risk signature and the tumour microenvironment and immunotherapy. Moreover, we performed a knockdown assay at the cellular level to verify the function of B4GALT5 in ovarian cancer, which was upregulated in ovarian tissues and associated with poor prognosis. The pathways involved were predicted by corresponding bioinformatic analyses.

## Methods

### Datasets collection

A total of 188 GT genes were recognized from the GlycoGene DataBase (GGDB, https://acgg.asia/ggdb2/). The RNA sequencing (RNA-seq) count data of these genes for OC (*N* = 420) and normal ovarian (*N* = 88) samples from the TCGA and GTEx were downloaded from UCSC Xena (https://toil.xenahubs.net). Relevant clinical information of 388 OC patients was obtained from TCGA (https://portal.gdc.cancer.gov/) database, including age, stage, grade, survival status and survival time. R software (version 4.1.3, https://www.r-project.org/) was used for further analysis.

An independent cohort (GSE26712) was downloaded from the GEO (http://www.ncbi.nlm.nih.gov/geo) database for validation, which contained 185 OC samples with expression profile and survival data. The clinicopathological features of OC patients from the TCGA and GEO databases are shown in Table 1.

**Table 1:** Clinicopathological features of OC patients enrolled in this study

Characteristics		TCGA database	GSE 26712
Total		388	185
Histological subtype		HGSOC	HGSOC
Age years	< 65 ≥ 65	252 137	-
Grade	G2 G3	47 341	-
FIGO Stage	II III IV	21 310 57	- 146 36
Follow-up months	< 1 1–120 > 120	8 377 3	1 173 11
Survival state	Deceased Living	218 170	56 129

Abbreviation: *HGSOC* High-Grade Serous Ovarian Cancer, *FIGO* International Federation of Gynecology and Obstetrics

### Determination and annotation of differentially expressed GTs in OC

The overall differentially expressed genes (DEGs) were identified by comparing the transcription data from the TCGA and GTEx databases using the R package “DEseq2”. The criteria of significance were *P*_adj_ < 0.05 and absolute log2FC > 1.5. Next, we converged the overall DEGs and GT genes as differentially expressed GT genes in OC. The functions of these genes were then determined by Gene Ontology (GO) and Kyoto Encyclopedia of Genes and Genomes (KEGG) analysis using the R package “clusterProfiler”. The correlation between these GTs was analysed using the “corrplot” package. A protein‒protein interaction (PPI) network was established by Search Tool for the Retrieval of Interacting Genes (STRING), version 11.5 (http://string-db.org/) with default minimum required interaction score of 0.4 (medium confidence) to explore physical and functional associations between DEGs. The network was clustered to a specified number of 3 using K-means clustering method. The ctyoHubba plugin in Cytoscape software (version 3.9.0) was used to find hub genes in PPI network.

### Construction and validation of the GT risk signature

Univariate Cox regression analyses were performed to assess the relationship between the differentially expressed GTs and overall survival (OS). Afterwards, LASSO Cox regression was employed to exclude colinear genes for fear of overfitting. Finally, a risk signature based on six GT genes (ALG8, B4GALT5, FUT8, ABO, ST6GAL1 and ST8SIA3) was identified. The coefficients of six hub genes obtained from the LASSO Cox were utilized to calculate the risk score according to the formula as follows:

$$\mathbf{R}\mathbf{i}\mathbf{s}\mathbf{k} \mathbf{s}\mathbf{c}\mathbf{o}\mathbf{r}\mathbf{e}\hspace{0.17em}= \sum_{{\varvec{i}}=1}^{{\varvec{n}}}{\varvec{C}}{\varvec{o}}{\varvec{e}}{{\varvec{f}}}_{{\varvec{i}}}*{{\varvec{x}}}_{{\varvec{i}}}$$

Where *Coef*_*i*_ and *x*_*i*_ represent the coefficients and expression level of each hub gene, respectively. Taking the median risk score as the cut-off value, OC patients were stratified into either a high-risk or a low-risk group. Furthermore, Kaplan‒Meier survival analysis was carried out to estimate the prognostic value by the R package “survival”. Receiver operating characteristic (ROC) curves were then used to check the performance of this prognostic prediction model in the “survival”, “survminer” and “timeROC” R packages.

The GSE26712 datasets served as the validation cohort to verify the prognostic value of this six-GT risk signature. The formula to determine the risk score and the cut-off criteria to classify the patients into a high-risk or a low-risk group were the same as the training cohort mentioned above.

### Correlations between the GT signature and clinical factors

Univariate and multivariate Cox regression analyses were used to determine whether the risk score played an independent prognostic role. Then, ROC curve analyses of the risk score and clinical parameters, including age, stage and grade, were performed to assess the prognostic value of this GT signature. A nomogram was established by the “rms” R package to predict the OS of individuals.

### Functional enrichment analysis of DEGs between the two subgroups

A total of 377 OC patients were stratified into high-risk and low-risk groups based on the median risk score. The DEGs between the two subgroups were singled out using the “DEseq2” R package with the screening threshold of log_2_FC > 1 and *P*_adj_ < 0.05. GO and KEGG analyses of these DEGs were performed by loading the R packages “clusterProfiler” and “GOplot”.

### Assessment of immune infiltration and immunotherapy efficiency

We used four algorithms to compare the immune infiltration status of two subgroups from the TCGA database in light of the expression profile of related genes. The Estimation of Stromal and Immune cells in Malignant Tumour tissues using Expression data (ESTIMATE) algorithm was utilized to count the Stromal Score, Immune Score and corresponding Estimate Score via the “estimate” R package. Then, CIBERSORT and TIMER R scripts were applied to calculate the proportions of TIICs (tumour immune infiltrating cells) in each sample. In the CIBERSORT algorithm, significant results (*P* < 0.05) were included for subsequent analysis. To quantify the 28 TIICs, we conducted a single-sample gene set enrichment analysis (ssGSEA) algorithm by inputting the expression matrix and immune cell marker gene set using the “GSVA” R package. The Wilcoxon rank-sum test was used to compare the content of infiltrating immune cells in OC between the low- and high-risk groups.

The Mutation Annotation Format (MAF) was downloaded from the TCGA database and analysed by the R package “maftools” to show the mutation landscape of the high- and low-risk groups. The number of somatic mutations and neoantigens were obtained from The Cancer Immunome Atlas (TCIA) (https://tcia.at). Immunophenoscore (IPS) analysis was performed to determine immunogenicity. IPS was calculated from the gene expression of typical cell types, the results of which were also obtained from TCIA. Furthermore, we took advantage of the Tumour Immune Dysfunction and Exclusion (TIDE) portal (http://tide.dfci.harvard.edu/) to predict the responsiveness to immune checkpoint blockade (ICB) therapy in both groups by retrieving the TIDE score, T-cell dysfunction score, and the infiltration level of myeloid-derived suppressor cells (MDSCs), tumour-associated fibroblasts (CAFs) and M2 tumour-associated macrophages (TAMs).

### Real-time quantitative PCR

Total RNA was purified by an RNA extraction kit (Thermo Scientific, USA) according to the manufacturer’s instructions. SYBR Green Mix (Vazyme, China) was then used by the ABI StepOnePlus Real-Time PCR machine (Applied Biosystems, USA) to perform Real-time quantitative PCR (RT‒qPCR). The primer sequences were as follows: B4GALT5, forward 5’-TACCGAGTTCTTTGGCGGAG-3’ and reverse 5’-AGCCTGCATTCTGTACTCTGTT-3’; GAPDH, forward 5’-GTCTCCTCTGACTTCAACAGCG-3’ and reverse 5’- AATGCCTTGGGCTTGCATCA -3’.

### Western blotting (WB) assay

Transfection efficiency was verified by WB assay. Briefly, the harvested cells were lysed using radioimmunoprecipitation assay (RIPA) lysis buffer (Beyotime, China) and a protein phosphatase inhibitor (Beyotime, China). The intact protein was separated by SDS‒PAGE and transferred onto a PVDF membrane using a wet transfer method. The membrane was blocked with 5% skim milk powder for 2 h at room temperature and then incubated at 4 °C overnight with primary antibodies specific for B4GALT5 (ab110398, Abcam, 1:1000 dilution, 45 KD) or alpha tubulin (1:1000, 55 KD); alpha tubulin served as the internal reference. Horseradish peroxidase (HRP)-labelled goat anti-rabbit IgG (Biosharp, 1:5000 dilution) was used as the secondary antibody. After incubating with the secondary antibody for 2 h at room temperature, the membrane was developed with an enhanced chemiluminescence (ECL) solution.

### Cell culture and transfection

A2780 human OC cells were cultured in high glucose DMEM (KeyGEN, China) supplemented with 10% foetal bovine serum (Gibco, USA) at 37 °C containing 5% CO_2_. Three siRNAs targeting the B4GALT5 mRNA region and Nc control siRNA were used for B4GALT5 silencing via transient transfection (RIBOBIO, China). The sequences of siRNAs against B4GALT5 were as follows: GTGGAACAATTTCGGAAAA (si-1); GATCGCAACTATTATGGAT (si-2); CAACCAAATTGGATAAGTA (si-3). Briefly, cells were seeded in a six-well plate 24 h before transfection. When cells reached 60%-70% confluence, Lipofectamine 3000 Transfection Reagent (Invitrogen, USA) was utilized to transfect with siRNAs following the manufacturer’s instructions. For validation, 48 h after transfection, total RNA and proteins were extracted for RT‒qPCR and WB assays as described above.

### Cell proliferation, invasion and migration assays

Cell proliferation assays were performed with a CCK-8 kit (Dojindo, Japan). Cells were harvested 24 h after transfection and then seeded into 96-well plates (3000 cells/well) with six replicates per sample. After 0, 24, 48 and 72 h, 100 µl of 10% CCK-8 serum-free medium was added. After 3 h of incubation, cell proliferation was estimated using a microplate reader (Bio-Tek, USA).

Cell migration and invasion were assessed using a 24-well Transwell plate (Corning, USA). For the migration assay, the upper chamber was filled with 200 μl serum-free medium and 5 × 10^4^ cells; the lower chamber was filled with 600 μl 20% FBS medium. After 24 h, the cells on the bottom surface were fixed with 4% paraformaldehyde and stained with crystal violet for 20 min. The migration assay was performed in the same manner but with Matrigel (Corning, USA) coated on the upper chamber.

### Gene set enrichment analysis of B4GALT5

A single-gene gene set enrichment analysis (GSEA) analysis was adopted to determine the molecular pathways associated with B4GALT5. The GSEA software (version 3.0) was downloaded from the official GSEA website (DOI:10.1073/pnas.0506580102, https://software.broadinstitute.org/gsea/index.gsp). The OC gene set from TCGA database was used for analysis and samples were divided into high expression group (≥ 50%) and low expression group (< 50%) based on the expression level of B4GALT5. The sub gene set “c2. cp. kegg.v.7.4. symbols. glmnt” was selected as the reference gene set. A pathway with *P* < 0.05 and *FDR* < 0.25 was considered as significant.

### Statistical analysis

R software (version 4.1.3) with the necessary packages, GraphPad Prism 9 and SangerBox platform were employed for all statistical analyses. The two-sample Wilcoxon rank-sum test and Kruskal‒Wallis test were performed for continuous data, and Pearson’s chi-square test and Fisher's exact test were performed for categorical data. For survival analysis, the log-rank test was used for KM analysis, and LASSO Cox proportional hazard regression was used to estimate the hazard ratios (HRs) and 95% confidence intervals (CIs). Unless otherwise specified, a 2-tailed *P* value less than 0.05 was considered statistically significant.

## Results

### Identification of differentially expressed GTs between normal and tumour tissues

**Fig. 2:**
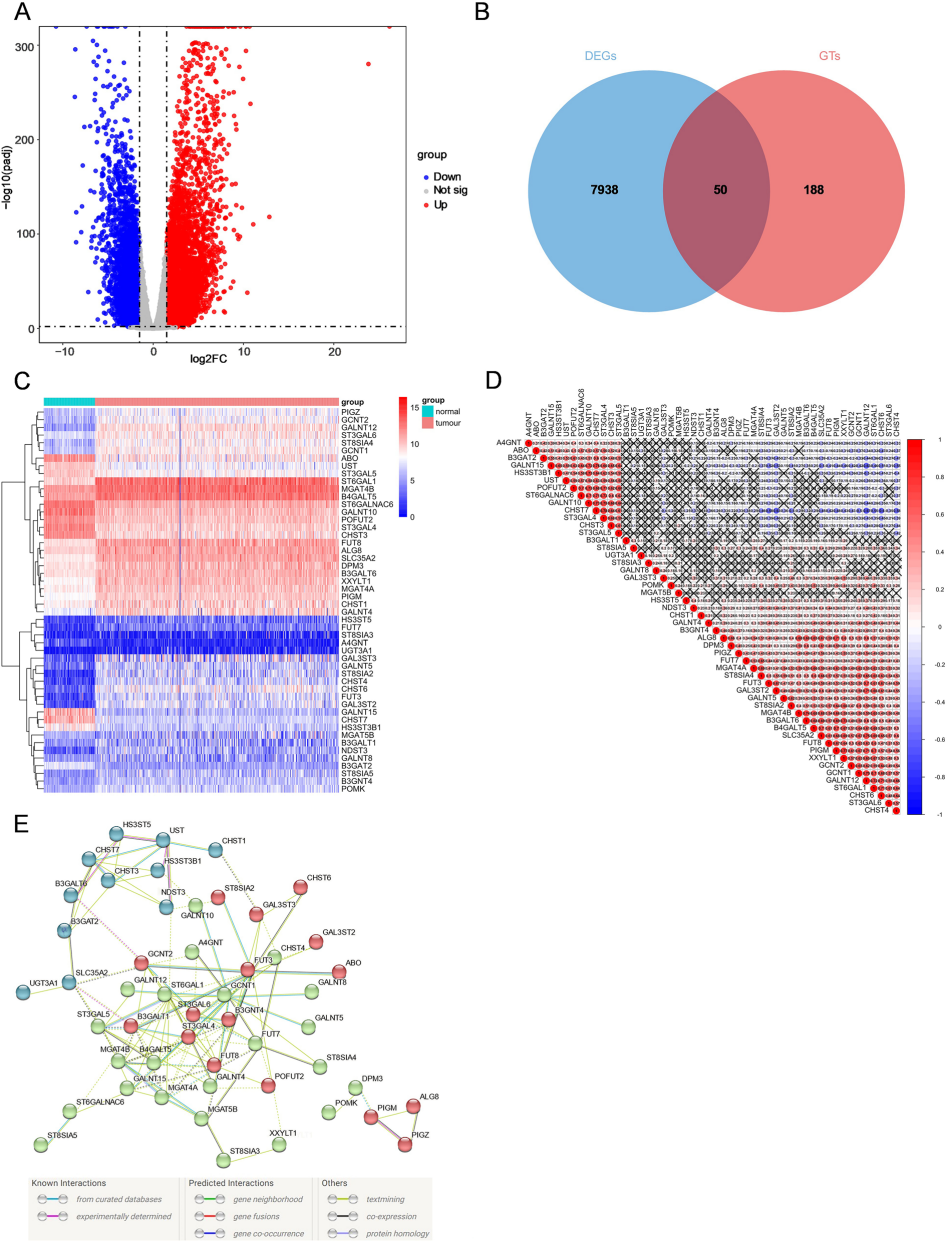
The expression profiling of GTs in ovarian cancer. (**A**) The volcano plot of DEGs between ovarian cancer and normal ovary tissues. The red and blue dots represent upregulated and downregulated genes respectively. (**B**) The Venn diagram for the intersections between DEGs and GTs. (**C**) Heatmap of 50 differentially expressed GTs in tumour and normal tissues. (**D**) Spearman’s correlation analysis of these 50 glycosyltransferase genes in ovarian cancer. (**E**) PPI network showing the interactions of differentially expressed GTs

A total of 7938 DEGs were identified by comparing data from 420 tumour and 88 normal tissues in the pooled TCGA OC and GTEx normal ovary datasets (Fig. 2A). By taking the intersection of OC DEGs and 188 GTs, we distinguished 50 differentially expressed GTs (Fig. 2B). Among them, 37 genes were upregulated, while 13 genes were downregulated in tumour tissues. The RNA levels of these genes are clearly shown in the heatmap (Fig. 2C). Subsequently, correlation analysis was conducted to further explore the associations between 50 GT genes. We found that CHST7 and ST3GAL4 were most positively relevant, while CHST7 and GALNT12 were most negatively relevant (Fig. 2D). To further explore the interaction between 50 GT genes, a PPI network was established and the colours of edges connecting two proteins represented different types of interactions including known, predicted or others (Fig. 2E). Among them, we determined that FUT8, B3GALT1, ST6GAL1, GCNT1, FUT3, ST3GAL4, MGAT4A, MGAT4B, B4GALT5, and ST3GAL6 were hub genes of the network. The functional analyses of 50 GTs are shown in Supplementary Fig. 1. These genes are involved in various types of protein glycosylation with the activity of glycosyltransferase.

### OC subgroups stratified by consensus clustering analysis of differentially expressed GTs

To investigate whether the expression of these 50 GTs plays a role in OC subtypes, we performed unsupervised consensus clustering of all 420 OC patients using the “ConsensusClusterPlus” R package. The optimum clustering variable (k = 2) was determined based on the comprehensive evaluation of several criteria (Supplementary Fig. 2A-B and Supplementary Fig. 3), indicating that the 420 OC patients were able to be divided into two clusters (Cluster 1 and Cluster 2). However, no significant difference in clinical features between Cluster 1 and Cluster 2 was observed. The OS time between the two clusters did not reach significance (*P* = 0.828, Supplementary Fig. 2C). To further clarify the relationship between clinical parameters, including grade, stage, age (< 65 or ≥ 65) and survival status, and two clusters, a heatmap with 50 GT expression levels of 388 patients who were equipped with complete clinical data was constructed (Supplementary Fig. 2D).

### Establishment of a prognostic risk model based on GTs in the TCGA training cohort

**Fig. 3:**
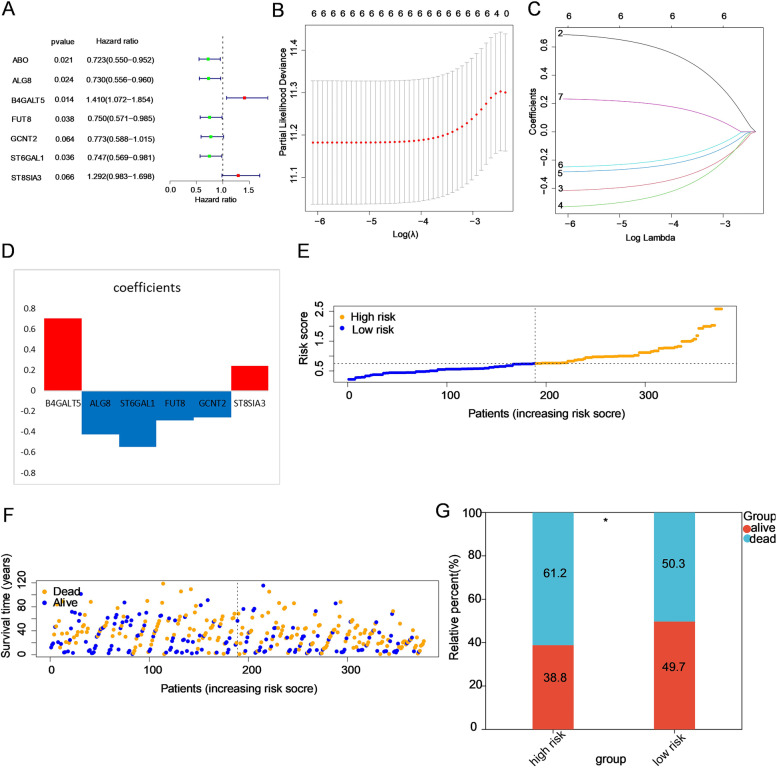
Construction of a prognostic risk model on the basis of these differentially expressed GTs. **A** Univariate Cox regression analysis of GTs. Factors with *P* < 0.1 are shown. **B-D** The process of establishing a risk signature with six glycosyltransferase genes. Coefficients were calculated by multivariate Cox regression by LASSO. **E** The distribution of risk scores in the prognostic model. **F** The distribution of survival status in the prognostic model. Patients in the high-risk group had more deaths and a shorter survival time than those in the low-risk group (the right side of the dotted line). **G** The proportion of deaths in two groups. *P < 0.05

A total of 377 samples (11 samples from patients whose OS time was < one month and > 10 years were excluded) with intact clinical data were used for subsequent risk signature construction. The genes associated with prognosis were preliminarily screened out by univariate Cox regression analysis. According to the threshold of *P* < 0.1, seven genes (ABO, ALG8, B4GALT5, FUT8, GCNT2, ST6GAL1 and ST8SIA3) were kept for further analysis, among which 2 genes (B4GALT5 and ST8SIA3) were considered risk factor genes with hazard ratios (HRs) > 1, and the other 5 genes (ABO, ALG8, FUT8, ST6GAL1 and GCNT2) were considered protective genes with HRs < 1 (Fig. 3A). The HR of a total of 50 genes is represented in Supplementary Fig. 3. Next, by performing LASSO Cox regression analysis, six genes (ALG8, B4GALT5, FUT8, GCNT2, ST6GAL1 and ST8SIA3) were selected to construct the risk model based on the minimum criteria (Fig. 3B-D). Accordingly, the risk scores of 377 patients were calculated by the coefficients of six genes, and patients were divided into high- and low-risk groups (Fig. 3E). The death rate of the low-risk group (50.3%) was lower than that of the high-risk group (61.2%) (*P* < 0.05, Fig. 3F-G).

**Fig. 4:**
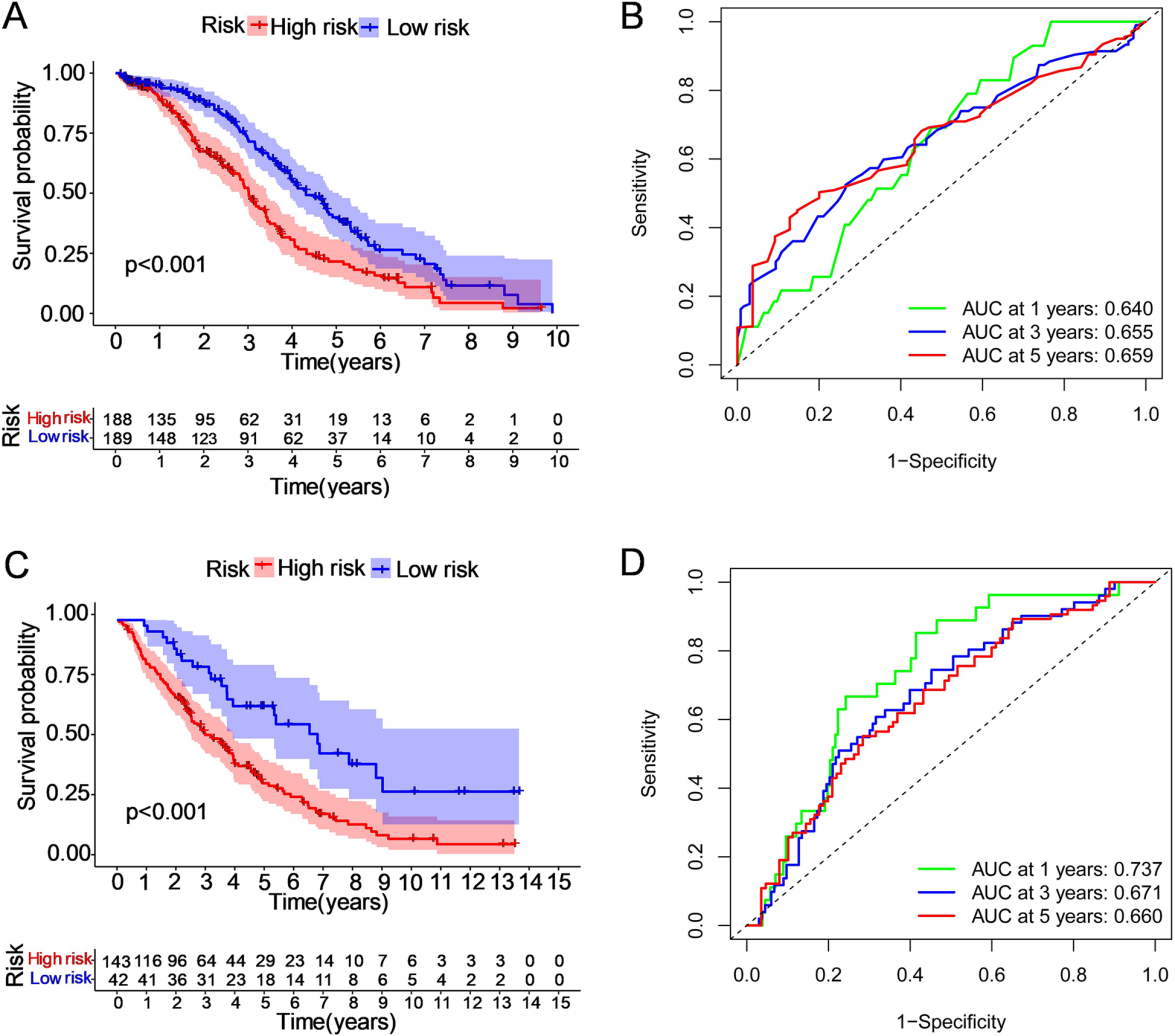
The Kaplan‒Meier OS analysis of prognostic models and the ROC curves showing the predictive efficiency of the risk signature. The patients in the two datasets were assigned to the high-risk and low-risk groups (separately represented by red and blue), taking the median risk score as the threshold. **A, B** In the TCGA discovery set, the survival rate of the high-risk group was lower than that of the low-risk group (*P *< 0.001). The areas under the curves (AUCs) at 1, 3, and 5 years were 0.640, 0.655, and 0.659, respectively. **C, D** In the GEO validation cohort, the survival rate was lower for the high-risk group than for the low-risk group (*P *< 0.001). The areas under the curve (AUCs) at 1, 3, and 5 years were 0.737, 0.671, and 0.660, respectively

Subsequently, we performed survival analysis of these two groups to probe the prognostic value of this six-gene risk model. The results showed that the OS of patients in the high-risk group was shorter than that of patients in the low-risk group (*P* = 1.15 × 10^–5^, Fig. 4A). We then drew receiver operating characteristic (ROC) curves to check the area under the curves (AUCs) at 1, 3, and 5 years of 0.640, 0.655, and 0.659, respectively, which demonstrated that the risk signature was reliable for predicting survival outcomes (Fig. 4B).

### Validation of the GT-based risk signature using the GEO database

 To validate the six-gene-based risk model, a total of 185 OC patients with prognostic information from the GEO database (GSE26712) were utilized as the external dataset. Patients were classified into low-risk (*N* = 93) and high-risk (*N* = 92) subgroups based on six risk-related GTs used in the TCGA cohort (Supplementary Fig. 5A). And the death rate of the low-risk group (52.4%) was lower than that of the high-risk group (74.8%) (Supplementary Fig. 5B-C). Consistent with the conclusions in the TCGA training cohort, the Kaplan‒Meier survival analysis demonstrated that the overall survival rate of the high-risk group was notably lower than that of the low-risk group (*P* = 7.14 × 10^–5^, Fig. 4C). The AUCs at 1, 3, and 5 years were 0.737, 0.671, and 0.660, respectively, indicating the superior efficiency of our model (Fig. 4D).

### Independent prognostic significance of this GT-based signature

**Fig. 5:**
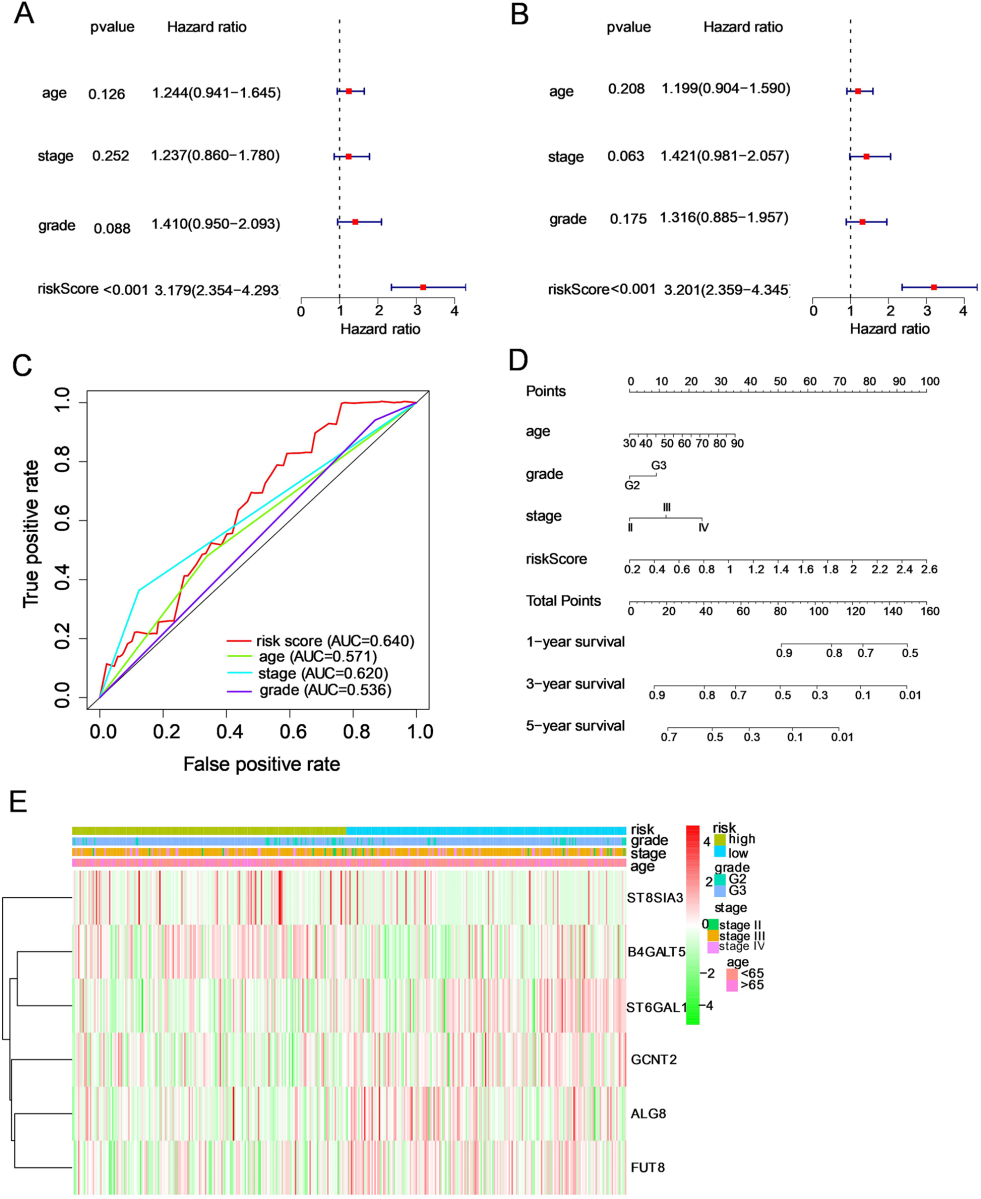
Effect of the risk signature combined with clinicopathological features on the prognosis of ovarian cancer patients. **A, B** Univariate and multivariate Cox regression analyses of the correlation between risk score plus clinicopathological features and overall survival. **C** The predictive ability of these factors was displayed by ROC curves. **D** Establishment of a prognostic nomogram for ovarian cancer patients by integrating the risk score and clinicopathological characteristics. **E** The expression of the six glycosyltransferase genes and distribution of clinicopathological characteristics between the two groups are shown by heatmap. When comparing the clinical parameters between the low- and high-risk groups, no significant differences were observed in terms of stage, grade or age

To explore whether this risk model was able to predict the prognosis of OC independently from clinicopathological features, we conducted univariate and multivariate Cox regression analyses to evaluate the associations between risk scores as well as clinical features and OS in OC patients. Univariate Cox regression analysis illustrated that the risk score could serve as a prognostic factor for OC (Fig. 5A, HR = 3.18 and* P* < 0.001). Then, after adjusting for confounding factors, the risk score was independently correlated with the OS of OC patients (Fig. 5B, HR = 3.20, *P* < 0.001). The ROC curves were plotted to define the predictive power of this signature. The AUCs for age, grade, stage, and risk score were 0.571, 0.536, 0.620, and 0.640, respectively, which meant that this model performed better in prognostic indicators than age, grade and stage (Fig. 5C). In addition, the expression levels of six genes in the high- and low-risk groups are displayed in a clustering heatmap (Fig. 5E). Finally, we built an innovative prognostic nomogram with age, grade, stage, and risk score to quantitatively predict the survival at 1, 3, and 5 years (Fig. 5D).

### Functional enrichment analysis on the basis of the risk signature

**Fig. 6:**
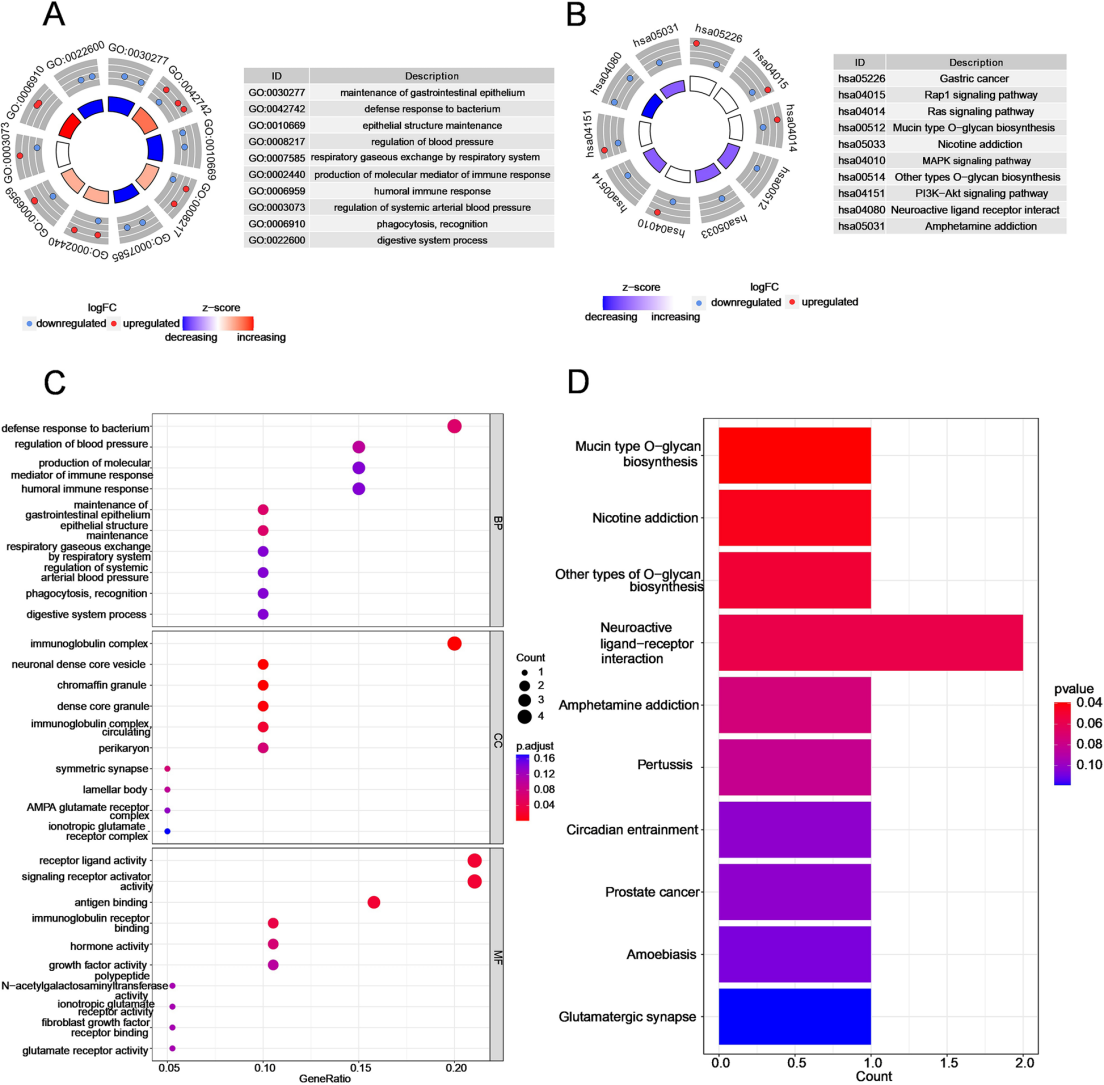
Functional analysis of DEGs between the two subgroups in the TCGA cohort. Circle plots of 10 enriched (**A**) GO terms and (**B**) KEGG pathways. The red and blue dots located in the outer rings represent upregulated and downregulated genes, respectively. The z score is portrayed by the colour of the inner ring. **C** Bubble plots for the top 10 biological processes (BPs), cellular components (CCs) and molecular functions (MFs) of GO enrichment. **D** Barplot graphs for the top 10 enriched KEGG pathways

To determine the potential mechanisms that lead to the apparently different clinical outcomes of the two subgroups stratified by this GT-based signature, we screened 38 DEGs between the high-risk and low-risk groups. Among them, 23 genes were downregulated, and the other 15 genes were upregulated (Supplementary Fig. 7). GO analysis revealed that these DEGs were mainly related to the immune response, immunoglobulin receptor binding and maintenance of epithelial structure (Fig. 6A and C). KEGG pathway analysis demonstrated that mucin-type O-glycan biosynthesis and several oncogenesis-related signalling pathways (such as the Raf1, Ras, MAPK and PI3K-Akt signalling pathways) were altered (Fig. 6B and D).

### Immune characterization of the two subgroups

**Fig. 7:**
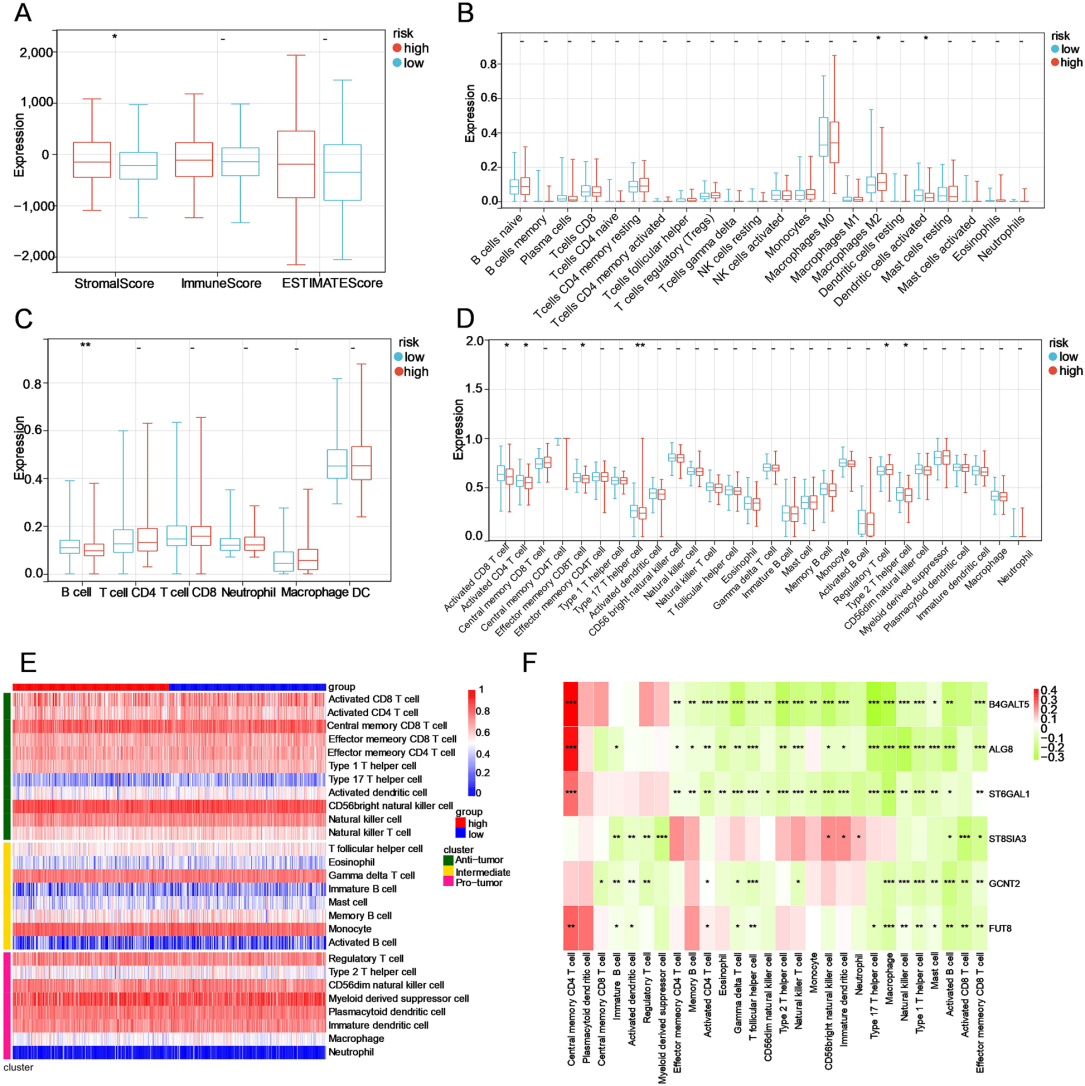
The immune infiltration landscape of the GT-based signature in OC. **A** Estimation of immune score, stromal score and ESTIMATE score between low- and high-risk patients using the ESTIMATE algorithm. **B, C** Evaluation of the proportions of immune cell subsets using the CIBERSORT and TIMER algorithms. (**D, E**) Boxplot and Heatmap of the infiltration levels of 28 immune cell subsets in the low- and high-risk groups calculated by ssGSEA, respectively. **F** Correlation heatmap between six hub genes and these 28 immune cell subdivisions. ***P < 0.001; **P < 0.01; *P < 0.05; -, not significant

Considering that this risk signature was connected with immune-related biological processes, several universally recognized methodologies were used to compute the immune cell infiltration score of samples in the two subgroups. The results of the ESTIMATE algorithm showed that the high-risk group had higher stromal scores and that the two subgroups exhibited no significant difference in immune scores, suggesting that nonimmune stromal cell infiltration significantly increased with increasing risk score (Fig. 7A). Based on the CIBERSORT algorithm, the high-risk group had a higher proportion of M2 macrophages, while the proportion of activated dendritic cells (DCs) was higher in the low-risk group (Fig. 7B). In the TIMER algorithm, B cells were more enriched in the low-risk group than in the high-risk group (Fig. 7C). Then, 28 types of IICs were evaluated by ssGSEA, the heatmap of which is depicted to show the distribution in the two groups (Fig. 7D). IICs of the antitumour cluster, including activated CD4 T cells, activated CD8 T cells, effector memory CD8 T cells, type 17 T helper cells and natural killer T cells, were abundant in the low-risk group, while regulatory T cells belonging to the protumour cluster were elevated in the high-risk group (Fig. 7E). Moreover, the correlation between the six hub genes and these 28 IICs is shown in a heatmap (Fig. 7F).

### Relationship between the risk signature and immunotherapy response

**Fig. 8:**
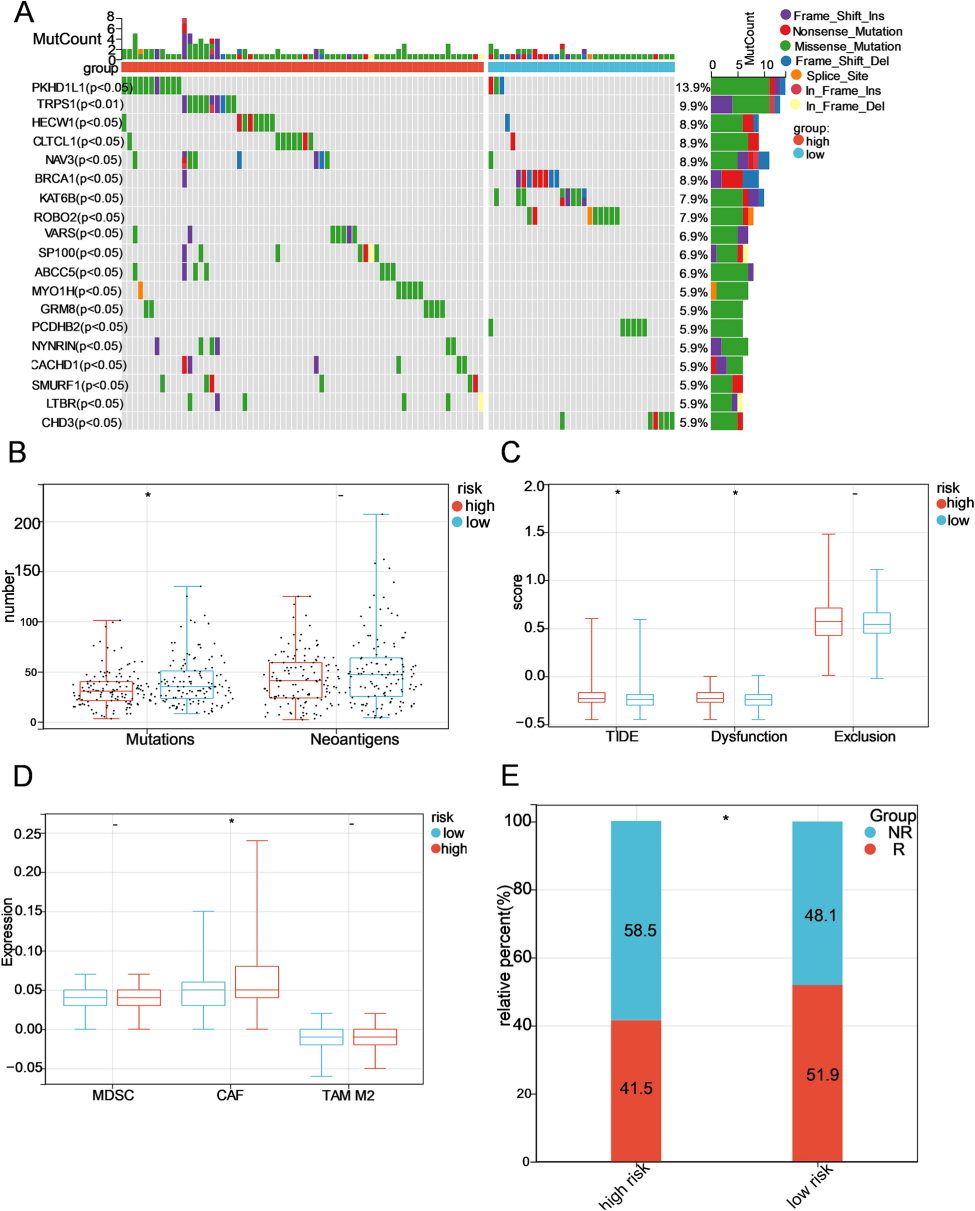
Identification of the GT-based signature for prediction of immune response in OC. **A** Waterfall plot of 19 genes with different mutation frequencies between the low- and high-risk groups. **B** The number of somatic mutations and neoantigens in low- and high-risk patients from the TCGA dataset. **C, D** The tide score, dysfunction score, exclusion score and infiltration level of MDSCs, CAFs, and M2 TAMs in the low- and high-risk groups. **E** The proportions of OC patients who responded to ICB in the low- and high-risk categories. R, responder; NR, nonresponder. *P < 0.05; -, not significant

Because mounting evidence has proven that somatic mutations may play a vital role in immunotherapy, we explored the mutational landscape of the two risk groups. The distribution of the top 20 frequently mutated genes was ranked, but none of them had a significant difference in the high-risk and low-risk groups (Supplementary Fig. 8A). We then analysed the distribution difference of somatic mutations between groups, and found that a total of 19 genes (PKHD1L1, TRPS1, HECW1, CLTCL1, NAV3, BRCA1, KAT6B, ROBO2, VARS, SP100, ABCC5, MYO1H, GRM8, PCDHB2, NYNRIN, CACHD1, SMURF1, LTBR, CHD3) had different mutation frequencies (except TRPS1 with *P* < 0.01, the rest with *P* < 0.05) (Fig. 8A). Moreover, the number of mutations in the low-risk group was greater than that in the high-risk group, while no significant association was found between the risk score and the number of neoantigens (Fig. 8B).

PD1 and CTLA4 were enrolled for IPS analysis, including four parts: ips_ctla4_neg_pd1_neg (CTLA4 negative response and PD1 negative response), ips_ctla4_neg_pd1_pos (CTLA4 negative response and PD1 positive response), ips_ctla4_pos_pd1_neg, and ips_ctla4_pos_pd1_pos. These four parts of the IPS showed no significant difference between the high- and low-risk groups (Supplementary Fig. 8B), which indicated that this GT-based signature might lack the ability to predict the immunophenotype. The TIDE score, a more accurate computing architecture consisting of dysfunction scores and exclusion scores, was used as a powerful predictor for ICB therapy. Patients in the high-risk group achieved a higher TIDE score and dysfunction score than those in the low-risk score group, while there was no significant difference in the exclusion score (Fig. 8C), illustrating that OC patients with higher risk scores were more prone to immune escape. In addition, CAFs, an important component of tumour microenvironment (TME) that may contribute to the formation of an immunosuppressive microenvironment, were evaluated in the high-risk group (Fig. 8D). According to the TIDE score, the low-risk group had more ICB therapy responder OC patients than the high-risk group (Fig. 8E).

### Knockdown assay of B4GALT5 in the proliferation, migration and invasion of OC cells

**Fig. 9:**
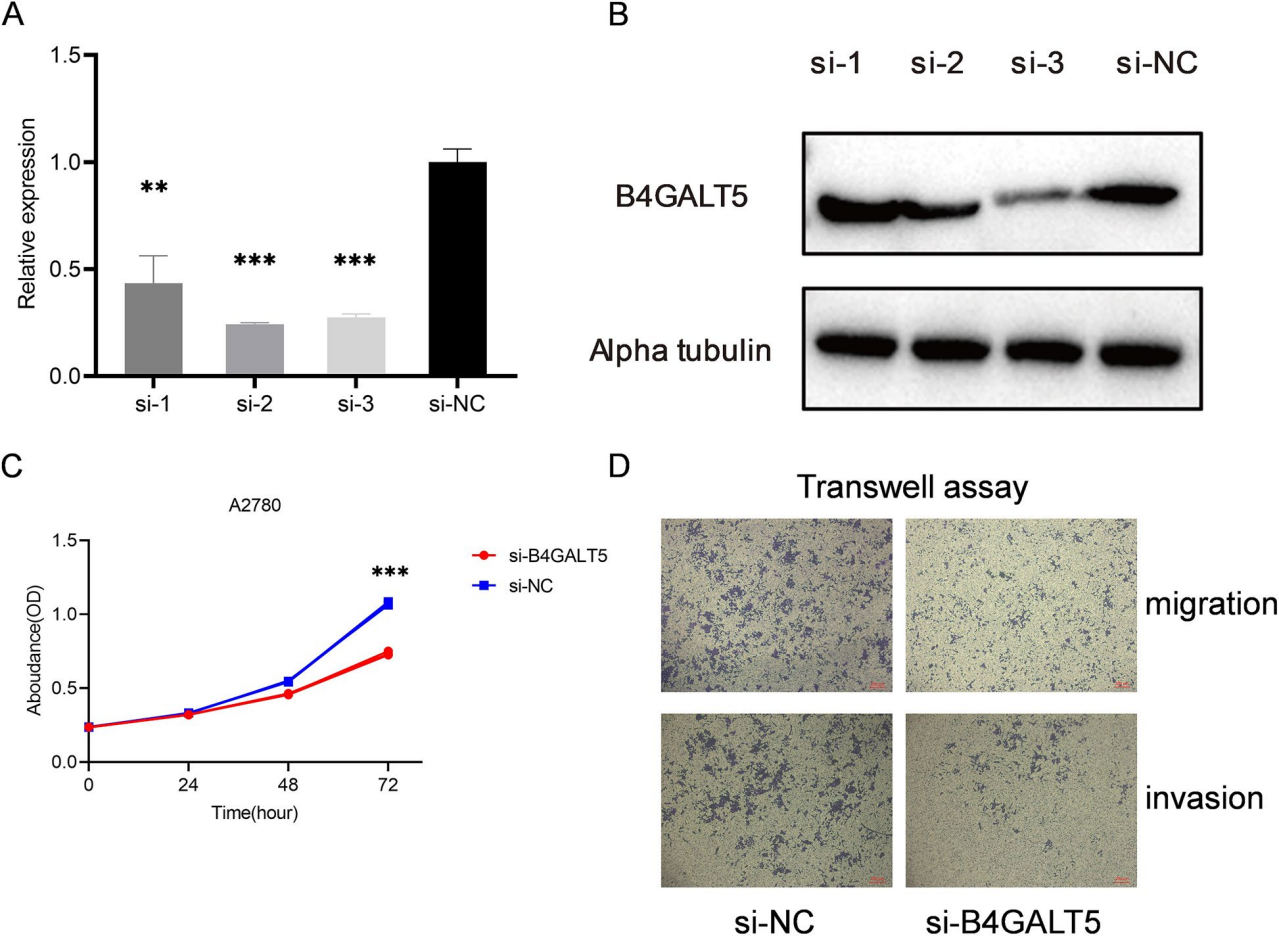
Knockdown of B4GALT5 affects the proliferation, migration and invasion of OC cells in vitro. **A, B** The transfection efficiency of B4GALT5 siRNA in OC cells evaluated by RT-qPCR and western blot. **C** Proliferation curve of the CCK8 assay between the knockdown and control groups. **D** Transwell assay results between the knockdown and control groups. **P < 0.01; ***P < 0.001

As the result of univariate Cox regression analysis showed (Fig. 3A), one of the genes (B4GALT5) was the risk factor (HR = 1.410, *P* = 0.014), and three of them (FUT8, ALG8, and ST6GAL1) were the protective factors (HR = 0.730, 0.750, 0.747 and *P* = 0.024, 0.038, 0.036, respectively). We then drew the Kaplan‒Meier OS curves of each gene, and the results were in accordance with the conclusion above (Supplementary Fig. 6B-G). The multivariate Cox regression analysis showed that B4GALT5 was the only independent risk factor (HR = 2.028, *P* < 0.001), and ALG8, ST6GAL1, FUT8 were independent protective factors with HR < 1 and *P* < 0.05 (Supplementary Fig. 6A). Among them, the upregulation of B4GALT5 in OC tissues compared to normal ovary tissues was logically consistent with the poor prognosis. Therefore, we further explored the function of B4GALT5 in OC cells, we transfected siRNA into OC cells to knock down B4GALT5 expression. The efficiency of transfection was then verified at the mRNA and protein levels using RT‒qPCR and WB assays, respectively (Fig. 9A-B). The CCK-8 assay showed that OC cells transfected with si-B4GALT5 exhibited significantly decreased proliferation compared with those in the control group (si-NC) (Fig. 9C). In the transwell assay, the knockdown of B4GALT5 significantly inhibited the migration and invasion of OC cells (Fig. 9D).

### Pathway prediction of B4GALT5 using bioinformatic analyses

**Fig. 10:**
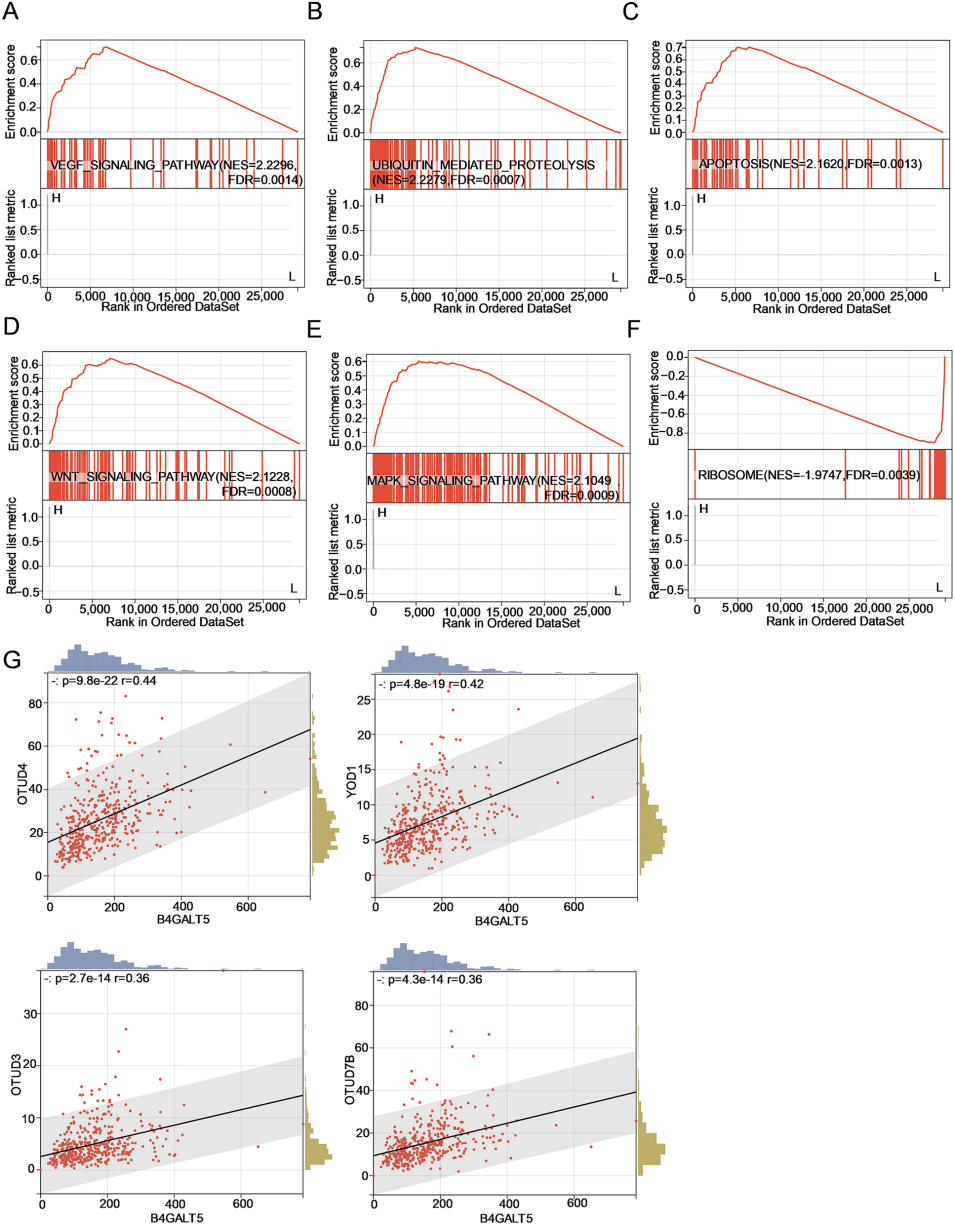
Pathway prediction and analysis of B4GALT5 in ovarian cancer. **A-F** Enrichment plots of DEGs between high- and low-expression levels of B4GALT5 from GSEA. **G** The relationship between the expression levels of OTU family deubiquitinases and B4GALT5. Scatter plots of four deubiquitinases with Pearson’s *r* > 0.3 and *P* < 0.05

To predict the pathways in which B4GALT5 is involved, we performed gene set enrichment analysis. The results showed that VEGF signalling pathway, ubiquitin-mediated proteolysis, apoptosis, WNT signalling pathway and MAPK signalling pathway were significantly enriched in high B4GALT5 expression group (Fig. 10A-E). And ribosome-associated pathway was enriched in low B4GALT5 expression group (Fig. 10F). Among them, ubiquitin-mediated proteolysis attracted our interest. Thus, Pearson correlation analysis between the expression level of B4GALT5 and OTU (ovarian tumour) family deubiquitinases (OTUB1, OTUB2, OTUD1, YOD1, OTUD3, OTUD4, OTUD7B) was performed. The results showed that four enzymes (YOD1, OTUD4, OTUD3 and OTUD7B) were positively correlated with B4GALT5 (*r* > 0.3 and *P* < 0.05), among which OTUD4 had the highest correlation coefficient of 0.44 (Fig. 10G).

## Discussion

Glycosylation, as an essential type of PTM, has aroused increasing interest in the academic community with recent advances in analytical technologies [28]. Although the intimate connection between glycosylation and cancer was detected decades ago [29–31], it has not been systematically researched in regards to GTs in OC until now. In the context of precision medicine, constructing a risk signature based on GT genes by means of machine learning made up for the insufficiency of one single factor to stratify OC patients. And we discussed the possibility that GT stratification together with different immune microenvironment will elucidate the poor prognosis of OC.

In our study, a total of 50 GT genes were differentially expressed in OC. However, the two clusters divided by the consensus clustering analysis based on these 50 DEGs showed little significant difference in clinicopathological characteristics. Encouragingly, a risk signature established on the basis of six GT DEGs (ALG8, B4GALT5, FUT8, GCNT2, ST6GAL1 and ST8SIA3) via Cox univariate analysis and LASSO Cox regression analysis was proven to predict the OS of OC patients effectively in the training and validation cohorts both with *P* < 0.001 and AUCs > 0.6. Additionally, the risk score was competent in predicting the prognosis for OC patients independently. A nomogram was successfully established; nonetheless, more studies should be conducted before clinical application.

FUT8 is an α-1,6-fucosyltransferase that take part in the core fucosylation of N-glycans and has a substrate specificity toward biantennary complex N-glycan oligosaccharide [32].But the sialylation of the N-glycans could reduce their activity as a substrate of FUT8 [33]. It was reported that there existed an interplay among FUT8, GnT-IV (MGAT4), and GnT-V (MGAT5) in N-linked glycosylation [34]. FUT8 was reported to be highly expressed in a variety of cancers, including lung, colorectal, ovarian, prostate, breast, melanoma and so on, and was associated with the prognosis of lung cancer, colorectal cancer and prostate cancer [15]. Overexpression of FUT8 can suppress the immune response in triple-negative breast cancer by mediating the abnormal N-glycosylation of B7H3, which may account for the lack of response to anti-PD1/PDL1 immunotherapy in triple-negative breast cancer patients [35]. In ovarian cancer, FUT8 activates the hyper core fucosylation of copper transporter 1 to suppress cisplatin uptake into OC cells [36]. However, FUT8 was found to be expressed at lower levels in osteosarcoma, leading to lower core fucosylation levels of TNF receptors. Lower fucosylation of TNF receptors decreased mitochondria-dependent apoptosis by activating NF-κB2 signalling [37]. Interestingly, our study revealed that FUT8 was upregulated in OC tissues but was a good prognostic factor. Similarly, ST6GAL1 was also overexpressed in OC but associated with favourable prognosis. The result was inconsistent with an earlier study where high ST6GAL1 protein expression in OC was significantly associated with poor prognosis [38]. ST6GAL1 is a β-galactoside α-2,6-sialyltransferase that catalyses α2,6-sialylation of N-glycans. It has been proved that FUT8-mediated core fucosylation of IgG limited the B cell-mediated IgG sialylation catalysed by ST6GAL1 [39, 40]. In view of the tight interaction between core fucosylation and α2,6-sialylation, we reasonably assume that the high sialylation level of the N-glycans in OC may affect the oncogenic function of FUT8. It is worth noting that ST6GAL1 was also found to be upregulated in many solid tumours [41–45] and promote their malignant phenotype by regulating the sialylation of signalling pathways [46]. What’s more, an increasing number of studies about the function of ST6GAL1 in immune enhancement have been published [47–51]. Thus, we also infer that the positive response to antitumour immunity may be another reason for its protective function in OC prognosis.

ST8SIA3 is another member of sialyltransferase family which is mainly involved in the sialylated glycolipids, while ST6GAL1 preferentially links α-2,6-linked sialic acid to Galβ4GlcNAc chains, usually present in N-linked chains [52]. Unlike ST6GAL1, there are relatively few studies focusing on the relationship between ST8SIA3 and cancer. ST8SIA3 was proved to participate in the synthesis of A2B5 epitope and was critical for A2B5 immunoreactivity in glioblastoma, indicating the potential of neuraminidase treatment [53]. Apart from that, other studies on ST8SIA3 were mainly about nervous system diseases and antiviral immunity [54, 55]. Interestingly, B4GALT5, another risk factor in our signature, was also reported to involve in the biosynthesis of lactosylceramide [56]. These results inspired us that the further study on the function of glycolipids in OC is promising. Except for glycolipids, B4GALT5 also acts with high preference on substrate that contain the GlcNAc beta1– > 6GalNAc structure which is found in mucin type O-linked core 6 glycan [57]. In our risk model, B4GALT5 was the GT gene with the highest coefficient of 0.7 and was the only independent risk factor, indicating that high levels of B4GALT5 might exert a stimulative effect on OC. In fact, many studies have elucidated its important role in many other tumour types. In colorectal cancer, B4GALT5 was considered as a diagnosis/prognostic biomarker with huge application potential which could be detected by electrochemical immunosensor [58]. In 2020, Tang et al. verified that B4GALT5 modulates the stemness of breast cancer through glycosylation modification to stabilize Frizzled-1 and activate Wnt/β-catenin signalling independent of its cell surface location [59]. A bioinformatic analysis concluded that B4GALT5 is one of the most consistently malignancy-associated enzymes using the transcriptomic data of the 21 TCGA cohorts [60]. Another recent bioinformatic analysis has drawn a similar conclusion as us regarding the negative role of B4GALT5 in hepatocellular carcinoma (HCC) prognosis and its vitro experiments also demonstrated that the knockdown of B4GALT5 in HCC cells was able to inhibit proliferation and metastasis [61], which is consistent with our in vitro experiments conducted on OC cells. In fact, about 20 years ago, researchers have found that beta1,4-galactosyltransferase was likely to be a biomarker in the monitoring OC patients when the serum CA 125 level is normal [62]. Taken together, B4GALT5 may be a promising starting point to help the mechanistic research of OC from the view of glycosylation.

GCNT2 is an initiated enzyme for the synthesis of I-branched glycan which is a cancer-associated glycan [63]. Loss of GCNT2 expression would lead to the ineffective I-branch conversion, thus regulating cancer progression. For example, high expression level of GCNT2 and its I-branched glycan product was proved to accelerate epithelial-to-mesenchymal transition in colon cancer [64]. GCNT2 has been widely studied in melanoma, the loss of which brings corresponding loss of I-antigen and thus enhances melanoma growth and metastasis [65]. Importantly, many studies have pointed out that the high level of I-antigen synthesized by GCNT2 could increase the susceptibility of malignant cells against immune cells in leukemia [66, 67]. This kind of immune regulatory may account for why GCNT2 was upregulated in OC but a good prognostic predictor in our study. What’s more, due to the role of β4GalTs in I-antigen capping, it’s really something to see that whether there exists an interaction between GCNT2 and B4GALT5. ALG8 is an alpha-1,3-glucosyltransferase and ALG8-CGD (congenital disorders of glycosylation) is a widely studied monogenic disorder of glycosylation that involves multisystem disorders [68]. In malignancies, ALG8 was a variate of a risk predictive model established for gastric cancer [69] and ovarian cancer [70]. However, consistent with Zhao et al.’s study [70], ALG8 in our study was overexpressed in OC tissues and led to favourable outcomes. In summary, except for ALG8, other five GTs are all key enzymes that involves in the synthesis of cancer associated glycans and may exist a close tie between each other.

Subsequently, the functional enrichment analyses demonstrated that DEGs between the two risk groups were correlated with immune responses and epithelial maintenance, supporting our speculations above. Ultimately, the immune cell infiltration in the low- and high-risk groups was compared, and we found that the low-risk group had increased infiltrating levels of antitumour immune cells in the TME, such as activated DCs [71, 72], B cells [73, 74], activated T cells [27, 75], and Type 17 T helper cells. In OC, T cells and B cells are associated with superior prognosis in ovarian cancer [76, 77]. High infiltration of type 17 T helper cells and DCs can also improve the outcomes of OC patients [78]. Simultaneously, high-risk patients had an immunosuppressive microenvironment with the presence of numerous CAFs and M2 TAMs. There is a popular belief that CAFs and M2 TAMs in the TME of OC are facilitators of carcinogenesis, tumour progression and metastasis, as well as therapeutic resistance and immunosuppression, leading to worse survival of OC patients [79–81]. Therefore, the GT-based signature established in our study is accurate enough to predict the OS of OC patients due to the consistent results with previous publications. Notably, GTs may provide a link to explore the crosstalk between cells in the TME and OC cells.

The highlight of our study is that the underlying role of this signature allows the prediction of patients who may benefit from ICB therapy. Genomic instability with accumulation of somatic mutations is widely believed to be associated with cancer cell phenotype shaping, thereby leading to different response to immunotherapy [82]. Tumour mutation burden (or the number of somatic mutations), which can give rise to neoantigens as targets of tumour immunity, has been explored as a promising biomarker for ICB therapy [83, 84]. Patients with high tumour mutation burden is more likely to benefit from ICB therapy and have an improved survival [85].Although the recognition of neoantigens is thought to be a random process, each somatic mutation is able to improve the opportunities for the immune system to recognize and attack cancer cells during ICBs [86, 87]. Our results showed that the number of somatic mutations in the low-risk group was higher than that in the high-risk group. Although not statistically significant, the neoantigens in low-risk group was higher, which, according to Lang F et al., may provide tumour-specific neoepitopes for individual therapeutical cancer vaccines [86]. We found that a total of 19 genes have significantly different mutational frequencies between two subgroups, which may provide supplementary targets for patients with poor response to ICB therapy. The high-risk group exhibited a higher TIDE score and dysfunction score than the low-risk group. The greater the TIDE score is, the less benefit patients achieve from ICB due to the more frequent existence of immune escape [88]. Meanwhile, according to the median TIDE score as the threshold of responders, the proportion of ICB responders was significantly higher in the low-risk group than in the high-risk group. Taken together, these results illustrated that the risk signature may be helpful in screening suitable candidates who would benefit more from ICBs.

The current study of GTs is still in its initial stages, especially regarding their mechanism in OC. Although our experiments have proven that B4GALT5 can regulate the progression of OC, the critical molecular mechanism is still unclear. Much effort will be put into probing the intrinsic mechanism, especially in determining whether B4GALT5 regulates ovarian cancer progression through abnormal glycosylation, in our subsequent studies. What is important to note is that although this signature was validated in an external dataset, formalin-fixed paraffin-embedded specimens and a multicentre prospective study are needed to confirm our findings.

## Conclusion

 In conclusion, we comprehensively studied the prognostic value of GT genes in OC and provided a theoretical foundation for future research. An effective prognostic signature was established based on six GT genes, and the risk score was an independent risk factor for OS prediction. Encouragingly, our results revealed that this signature may be a predictor of ICB response, contributing to the advancement of precision medicine. Moreover, the preliminary experiment revealed the promoting function of B4GALT5 in OC progression, and bioinformatic analysis predicted the likely pathways involved, paving the way for our follow-up study.

## Supplementary Information

**Additional file 1:** **Supplementary figure 1. **Functional annotation of fifty differentially expressed glycosyltransferase genes (GTs) in ovarian cancer. (A-C) Functional annotation using Gene Ontology (GO) terms (including Biological Process, Cellular Component and Molecular Function). (D) Barplot graph of the top ten enriched pathways using KEGG analysis.

**Additional file 2:** **Supplementary figure 2.** OC classification based on these differentially expressed genes. (A) Consensus clustering tracking plot for k = 2 to 9. (B) Consensus matrix for k = 2, in line with which 420 OC patients were grouped into two clusters. (C) Kaplan‒Meier overall survival curves for patients in Clusters 1 and 2 (samples with a survival time of more than ten years and less than one month were excluded). (D) Heatmap and clinicopathological characteristics of these two clusters. Red represents high expression, and blue represents low expression.

**Additional file 3:** **Supplementary figure 3.** Consensus clustering of OC. (A) The cumulative distribution function (CDF) is displayed for k = 2–9. (B) The curve of the relative change in the area under the CDF for k = 2 to 9. (C-I) Consensus clustering matrix for k = 3 to 9.

**Additional file 4:** **Supplementary figure 4. **Forest plot of 50 differentially expressed GTs using univariate Cox regression analysis based on the data from the TCGA.

**Additional file 5:** **Supplementary figure 5. **Construction of the risk model in the GEO dataset for validation. (A) The distribution of risk scores in the prognostic model. (B) The distribution of survival status in the prognostic model. (C) The proportion of deaths in two groups. **P < 0.01.

**Additional file 6:** **Supplementary figure 6. **Survival analysis of six GT genes. (A) Forest plot of six GT genes using multivariate Cox regression analysis. (B-G) KM survival plotter curves of the six hub genes.

**Additional file 7:** **Supplementary figure 7.** The volcano plot of DEGs between the high-risk and low-risk groups. The red dots represent upregulated genes, and the blue dots represent downregulated genes.

**Additional file 8:** **Supplementary figure 8. **(A) Waterfall plot of the top 20 somatic mutations in the low- and high-risk groups of the TCGA dataset. (B) IPS value of four parts in two subgroups.

## Data Availability

The from this study is available from the corresponding author upon reasonable request.

## Abbreviations

OC: Ovarian cancer
GTs: Glycosyltransferases
ICBs: Immune checkpoint blockade therapies
TCGA: The Cancer Genome Atlas
GTEx: Genotype-Tissue Expression
GEO: Gene Expression Omnibus
LASSO: The least absolute shrinkage and selection operator
PTMs: Post-translational modifications
DEGs: Differentially expressed genes
GGDB: GlycoGene DataBase
HR: Hazard ratios
OS: Overall survival
CI: Confidence interval
ROC: Receiver operating characteristic
GSEA: Gene set enrichment analysis
TIICs: Tumour immune infiltration cells
TME: Tumour microenvironment
TAMs: Tumour associated macrophages
CAFs: Cancer associated fibroblasts
TCIA: The Cancer Immunome Atlas
IPS: Immunophenoscore
TIDE: Tumour Immune Dysfunction and Exclusion
