# Supplementary figures and images for: A signature based on glycosyltransferase genes provides a promising tool for the prediction of prognosis and immunotherapy responsiveness in ovarian cancer
Source: J Ovarian Res. 2023 Jan 7;16:5. doi: 10.1186/s13048-022-01088-9 (PMC9826597; doi:10.1186/s13048-022-01088-9)

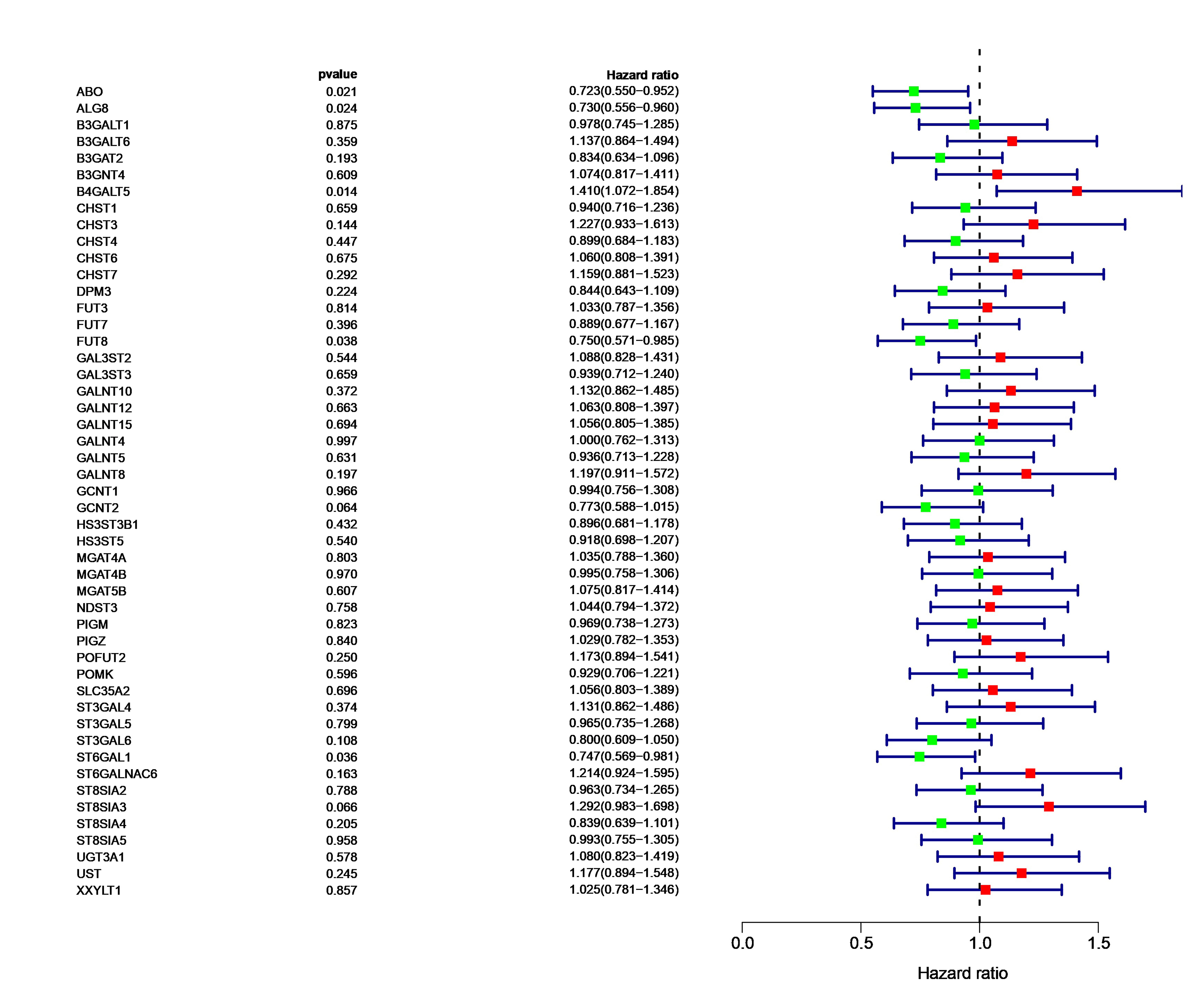

Supplement: Supplementary file 4 — Additional file 4: Supplementary figure 4. Forest plot of 50 differentially expressed GTs using univariate Cox regression analysis based on the data from the TCGA. [file 13048_2022_1088_MOESM4_ESM.jpg]

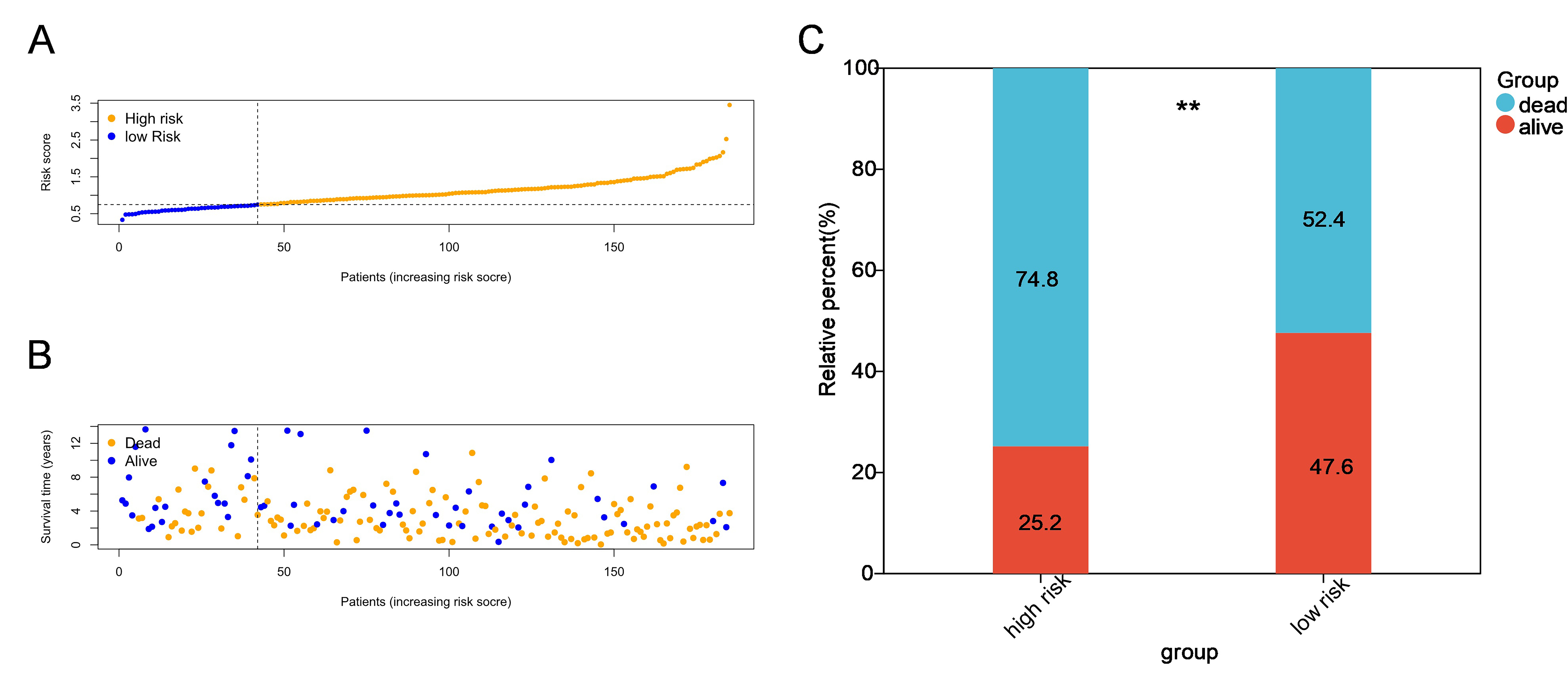

Supplement: Supplementary file 5 — Additional file 5: Supplementary figure 5. Construction of the risk model in the GEO dataset for validation. (A) The distribution of risk scores in the prognostic model. (B) The distribution of survival status in the prognostic model. (C) The proportion of deaths in two groups. **P < 0.01. [file 13048_2022_1088_MOESM5_ESM.jpg]
